# Fluorogenic Biosensing with Tunable Polydiacetylene Vesicles

**DOI:** 10.3390/bios15010027

**Published:** 2025-01-07

**Authors:** John S. Miller, Tanner J. Finney, Ethan Ilagan, Skye Frank, Ye Chen-Izu, Keishi Suga, Tonya L. Kuhl

**Affiliations:** 1 Department of Materials Science and Engineering, University of California Davis, Davis, CA 95616, USA; jsmiller@ucdavis.edu; 2 Department of Chemical Engineering, University of California Davis, Davis, CA 95616, USA; tjfinney@lanl.gov (T.J.F.);; 3 Materials Synthesis and Integrated Devices Group, Los Alamos National Laboratory, Materials Physics and Applications Division, Los Alamos, NM 87545, USA; 4 Department of Biomedical Engineering, University of California Davis, Davis, CA 95616, USA; ychenizu@ucdavis.edu; 5 Department of Pharmacology, University of California Davis, Davis, CA 95616, USA; 6 Department of Internal Medicine/Cardiology, University of California Davis, Davis, CA 95616, USA; 7 Department of Chemical Engineering, Graduate School of Engineering, Tohoku University, Sendai 980-8579, Miyagi, Japan

**Keywords:** biosensing, polydiacetylene, vesicles, fluorescence, spectroscopy

## Abstract

Polydiacetylenes (PDAs) are conjugated polymers that are well known for their colorimetric transition from blue to red with the application of energetic stimulus. Sensing platforms based on polymerized diacetylene surfactant vesicles and other structures have been widely demonstrated for various colorimetric biosensing applications. Although less studied and utilized, the transition also results in a change from a non-fluorescent to a highly fluorescent state, making polydiacetylenes useful for both colorimetric and fluorogenic sensing applications. Here, we focus on the characterization and optimization of polydiacetylene vesicles to tune their sensitivity for fluorogenic sensing applications. Particularly, we look at how the structure of the diacetylene (DA) hydrocarbon tail and headgroup affect the self-assembled vesicle size and stability, polymerization kinetics, and the fluorogenic, blue to red phase transition. Longer DA acyl tails generally resulted in smaller and more stable vesicles. The polymerization kinetics and the blue to red transition were a function of both the DA acyl tail length and structure of the headgroup. Decreasing the acyl tail length generally led to vesicles that were more sensitive to energetic stimuli. Headgroup modifications had different effects depending on the structure of the headgroup. Ethanolamine headgroups resulted in vesicles with potentially increased stimuli responsivity. The lower energy stimulus to induce the chromatic transition was attributed to an increase in headgroup hydrogen bonding and polymer backbone strain. Boronic-acid headgroup functionalization led to vesicles that were generally unstable, only weakly polymerized, and unable to fully transform to the red phase due to strong polar, aromatic headgroup interactions. This work presents the design of PDA vesicles in the context of biosensing platforms and includes a discussion of the past, present, and future of PDA biosensing.

## 1. Introduction

Polydiacetylenes (PDAs) are conjugated, linear polymers with vibrant chromatic properties. Diacetylene (DA) monomers undergo a 1,4 addition topochemical polymerization reaction when the monomers pack into a correct geometry. The resulting polymer is visibly blue with an alternating double-triple-bond (ene-yne) motif that forms the polymer backbone. The blue phase can transform into a visibly red phase polymer with the application of some additional stimulus (light, heat, and mechanical stress), which perturbs the polymer backbone and its conjugation state. The blue phase is generally non-fluorescent, while the red phase is highly fluorescent, making PDAs useful as both colorimetric and fluorogenic sensors [1,2]. A large body of work has utilized the colorimetric properties of PDAs for non-specific sensors of temperature, pH, and solvent exposure. The earliest demonstrated sensors were for bio-specific sensing. For example, Charych et al. [3,4] utilized polydiacetylenes for sensing the presence of influenza virus. Biospecificity was conferred by functionalizing the diacetylene surfactant carboxylic headgroup with sialic acid, which binds to hemagglutinin on the surface of influenza virus. They then took advantage of the DA surfactant properties to fabricate a colorimetric biosensor film. The hemagglutinin–sialic acid receptor–ligand interaction was sufficient to perturb the polymer backbone and drive the transition from the blue to red phase [3,4]. Since this demonstration, the polydiacetylene sensing community has exploded and PDA-based sensors have been highlighted in several notable reviews [5,6,7,8,9,10,11,12]. Some select PDA biosensor applications that span receptor–ligand interactions, molecular adhesion, and cell motility are summarized in Table 1. Improved sensor designs are expanding the application space.

PDA biosensors are typically fabricated from commercially available diacetylene surfactants. These surfactants have a carboxylic acid headgroup and a long hydrocarbon tail (>C17) with a diacetylene motif (two consecutive triple bonds) usually starting at the 10 carbon in the acyl chain. As with typical surfactants, the diacetylene surfactants spontaneously self-assemble into various structures in water to minimize their free energy. Micelles, fibers, and Langmuir films have been used as biosensors, but the most prevalent are vesicles [3,4,19,20,21]. PDA vesicles have been made by thin film hydration, microfluidics, and solvent injection [4,22,23,24,25,26]. Vesicles can also be embedded into a larger matrix structure like a hydrogel, fiber, polymer composite, or silica composite to create a sensing platform [27,28,29,30,31,32,33].

The focus of most PDA sensing literature is on engineering the surfactant-based sensors for a desired application. Generally, this includes modifying the DA surfactant with a ligand that interacts with a receptor on a surface or free in solution or by mixing some biological lipids (phospholipids or cholesterol) into the polymer matrix to interact with some analyte in solution [3,4,34,35]. The binding of the analyte perturbs the polymer backbone and drives the blue to red transition. In biosensing, tailoring vesicle sensitivity is important to target a range of biochemical or biophysical interactions that are generally weak but specific. The two primary pathways for tuning the properties of polydiacetylene vesicles include changing the solution conditions for the self-assembly or changing the structure of the surfactant. These procedures typically either weaken the intermolecular forces between surfactant molecules to make the transition easier or strengthen the intermolecular interactions to make the transition more difficult. Tailoring the solution conditions can include changing the solution pH, ionic strength or using additives such as alcohols or phospholipids that affect molecular packing in the bilayer [36,37,38,39,40]. Modifying the surfactant structure typically involves changing the length of the acyl spacer between the headgroup and diacetylene motif, changing the structure of the headgroup, or changing the length of the alkyl tail behind the diacetylene motif to tailor the intermolecular interactions between neighboring surfactants [21,22,24,25,41,42]. In all cases, the design of vesicles for specific applications requires some degree of optimization to ensure topochemical polymerization, as the structure of a diacetylene surfactant directly influences its assembled size, polymerization properties, and sensitivity. Traiphol et al. investigated how the headgroup, alkyl spacer length, and alkyl tail of PDA vesicles affected their thermal, pH, and solvent sensitivities in the context of designing colorimetric sensors [24,25,41,42]. Chandra et al. also investigated the polymerization and colorimetric responses of PDAs as a function of alkyl tail and spacer length using vesicles synthesized from a solvent injection approach [22,23]. Various other authors have looked at modifying polydiacetylenes with either amino acids or other supramolecules to tailor their sensitivity to external stimuli [21,43,44,45].

**Figure 1:**
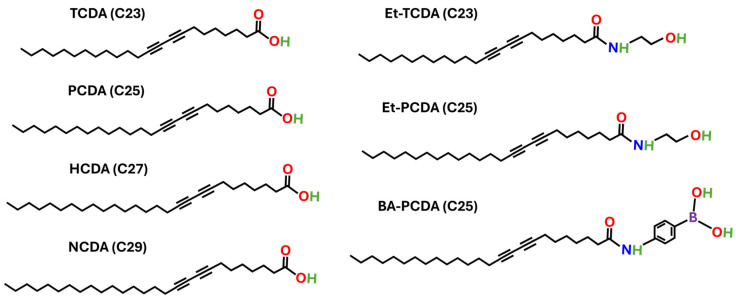
Structures of diacetylene surfactants used in this study including: 10,12 tricosadiynoic acid (TCDA), 10,12 pentacosadiynoic acid (PCDA), 10,12 heptacosadiynoic acid (HCDA), 10,12 nonacosadiynoic acid (NCDA), ethanolamine functionalized tricosadiynoic acid (Et-TCDA), ethanolamine functionalized pentacosadiynoic acid (Et-PCDA), and boronic acid functionalized pentacosadiynoic acid (BA-PCDA).

Here, we focus on building a framework for optimizing and developing PDA vesicles that can then be used as a broad-spectrum biosensing platform. PDA vesicles with different chain lengths (C23–C29) and different headgroups (carboxylic acid, ethanolamine, and boronic acid) were synthesized by a solvent injection approach and characterized for size, polymerization kinetics, blue–red transition kinetics, fluorescence transition characteristics, and biological sensing responsivity to arginine as an exemplar. Figure 1 shows the different surfactants utilized in this study. The goal of this paper is to provide readers with strategies for tailoring the stability and sensitivity of polydiacetylene vesicles as the foundation for any desired biosensing application.

## 2. Materials and Methods

### 2.1. Surfactant Preparation

Diacetylene (DA) surfactants were purchased from TCI Chemicals (Portland, OR, USA), GFS Chemicals (Powell, OH, USA), and Wako Fine Chemicals (Richmond, VA, USA). Surfactants used for synthesis include 10,12 tricosadiynoic acid (TCDA); 10,12 pentacosadiynoic acid (PCDA); 10,12 heptacosadiynoic acid (HCDA); and 10,12 nonacosadiynoic acid (NCDA). Reagents were from Sigma Adrich (St. Louis, MO, USA), GFS Fine chemicals (Powell, OH, USA), TCI Chemicals (Portland, OR, USA), and Boron Molecular (Raleigh, NC, USA). MilliQ water was from a house Barnstead Nanopure (Waltham, MA, USA) filtration source (18 MOhms).

Diacetylene surfactants commercially purchased typically have a high fraction of polymerized blue phase that should be removed prior to use. Silica gel chromatography was used to separate PDA monomer content from its polymer content using chloroform as the mobile phase following [46]. Chloroform fractions containing monomers were combined and dried using a rotary evaporator at 30 °C. Dried powder samples were placed in amber vials, covered in aluminum foil, and stored in a −20 °C freezer to minimize undesirable polymerization reactions. Other researchers have documented syringe filtering for the separation of the polymer content from diacetylene monomers, but this does not remove all polymer content as effectively as chromatography [25,46].

### 2.2. Ethanolamine Functionalized Diacetylene Surfactants

Diacetylene surfactants of varying alkyl tail lengths between C17 and C29 can be commercially purchased. Surfactants with varying headgroup structures are not as widely available. The carboxylic headgroup on fatty acid diacetylene surfactants can be easily modified using peptide coupling procedures. In this work, PCDA and TCDA were functionalized with ethanolamine using the N-hydroxy succinimide (NHS) and 1-ethyl-3-(3-dimethylaminopropyl)carbodiimide (EDC) coupling scheme. In this procedure, 1.1 equivalents of NHS and EDC were mixed with a desired mass of the diacetylene surfactant (typically ~100 mg), dissolved in dichloromethane (DCM), and stirred overnight. The DCM was then removed utilizing roto-evaporation at room temperature. The dried product was dissolved in ethyl acetate, and an ethyl acetate/water extraction was used to remove the reaction byproducts and unreacted material. The product solution was dried over magnesium sulfate and separated from the magnesium sulfate using gravity filtration through Whatman 1 filter paper (St. Louis, MO, USA) and a separatory funnel. The ethyl acetate was then removed using a roto-evaporator (Buchi New Castle, DE, USA) at 40 °C. The product was an NHS-diacetylene surfactant which was used right away to avoid unwanted polymerization of the surfactant. NHS-diacetylene was mixed with 1.1 equivalents of ethanolamine for functionalization, triethyl amine (TEA) to maintain a basic pH environment, and DCM. The mixture was stirred and allowed to react overnight. DCM was again removed by roto-evaporation. The product was reconstituted using ethyl acetate and separated using an ethyl acetate/water extraction and brine wash. The product was dried using magnesium sulfate, collected by gravity filtration over Whatman 1 filter paper, and the solvent removed by rotary evaporation. Samples were placed in amber vials and stored at −20 °C. The successful coupling of precursors was validated with nuclear magnetic resonance (NMR, see Supplementary Materials (Figures S1 and S2).

### 2.3. Boronic Acid Functionalized Diacetylene Surfactants

Boronic acid functionalized PCDA was made using a one-pot synthesis approach. The desired amount of PCDA (~50–100 mg) was dissolved in dimethyl formamide (DMF) in a single pot with 1.1 equivalents of 4-aminophenyl boronic acid, 1.1 equivalents of EDC, 1.1 equivalents of 1-hydroxy-benzotriazole (HOBt), and 4 equivalents of TEA. The solution was stirred overnight in a clean glass vial covered in aluminum foil. After one day, water was added dropwise to the DMF solution to precipitate the product. The product was collected using a vacuum filtration set-up over Whatman 1 filter paper. The product was washed three times using ethyl acetate and water, followed by a brine and sodium bicarbonate wash to remove unreacted reagents and byproducts. Samples were dried overnight to remove any adsorbed solvent or water. Product formation was verified using an alizarin red dye test (see Supplementary Figure S3).

### 2.4. Vesicle Synthesis, Preparation, and Polymerization

Diacetylene vesicles were synthesized using a solvent injection process [22,23]. Purified DA surfactants were dispersed in ethanol to make a 2 mg/mL solution. Approximately 500 µL of solution was slowly injected into 15 mL of heated water and vigorously stirred for approximately 1 h. The water was heated 5–10 °C above the respective surfactant melting temperature. Surfactant concentrations in solution ranged from 0.12 to 0.18 mM. After heated stirring, the vesicle solution was refrigerated overnight at 4 °C to allow for self-assembly and the topochemical arrangement for polymerization.

For polymerization studies, 3 mL samples were extracted and transferred into a glass cuvette. Samples were polymerized using two UVC (254 nm) pen lamps (Spectroline Melville, NY, USA) on each side of the cuvette. A schematic of the polymerization set-up and reaction scheme is provided in the Supplementary Materials (Figure S4). The UV irradiance on the cuvettes was calibrated to be 9.6 ± 0.9 mW/cm^2^ using a light meter (G&R Labs, Model 200, Santa Cruz, CA, USA). For photochromism studies, samples were exposed to UV light and 250 µL samples were taken at discrete time intervals, which correspond to a quantifiable UV dose. For dynamic light scattering, thermochromism, and bio-chromism studies, samples were polymerized to their optimal blue, filtered through a 5 µm filter to removed aggregated polymer content, and diluted by 50% for measurements. More details on the characterization methodologies are provided in the next section.

### 2.5. Vesicle Characterization

Dynamic light scattering (DLS) was utilized to measure the average particle sizes of PDA vesicles using a Brookhaven 90Plus Particle Size Analyzer (Nashua, NH, USA). Results from three independent samples were averaged. Two samples per vial were taken for a total of six measurements. Intensity-weighted average particle sizes and dispersity from log normal distributions are reported.

The optical properties of PDA vesicles were obtained by both UV–visible absorption spectroscopy and fluorescence spectroscopy. UV–vis absorption measurements were taken using a Spectromax M4 spectrophotometer (Molecular Devices, San Jose, CA, USA) using a well plate reader. Absorbance spectra measurements for blue and red phase vesicles were taken from 400 to 700 nm in 1 nm increments in quartz cuvettes with a 10 mm optical path length. Absorption spectra for UV-induced polymerization studies were taken from 400 to 700 nm in 2 nm increments using a 96 well plate reader. The evolution of the blue and red peaks was directly tracked to monitor the evolution of the blue and red phases and measure the UV-induced blue to red transition kinetics by fitting the blue phase decay to a first-order exponential decay. Spectra-integrated intensity measurements were utilized to characterize the polymerization kinetics of the monomer to polymer PDA phases as a function of increasing UV dose by fitting to a first-order model. Fluorescence spectroscopy was used to measure the fluorescence spectra of the red phase with a Jasco 8500 Fluorimeter (Easton, MD, USA) using a 490 nm excitation wavelength and a 525 and 700 nm spectral range in 1 nm increments. For thermal fluorescence measurements, the samples were heated in a glass cuvette at 5 °C increments from room temperature to 95 °C. The solution was excited at 490 nm and emission at 560 nm was measured.

Biosensing was demonstrated with PDAs by screening for the amino acid arginine using the Spectromax M4 spectrophotometer well plate reader in both fluorescence and absorbance. Samples of Et-PCDA, PCDA, and NCDA vesicles were screened against solutions containing arginine. Two hundred microliters of vesicle solution were added to a 96 well plate reader and mixed with 50 μL of varying concentrations of arginine to provide molar equivalents to PDA ranging from 0 to 10. The colorimetric response (*CR*) from the absorption was taken by measuring the change in percent blue phase given by Equations (1) and (2), where *PB_o_* is the initial percent blue phase, *PB* is the measured percent blue phase, *A_R_* is peak absorbance of the red phase, and *A_B_* is the peak absorbance of the blue phase [3]. The fluorescence was measured with an excitation wavelength of 485 nm and an emission wavelength of 555 nm. The change in fluorescence from the measured to initial fluorescence is reported.

(1) $$CR=\frac{{PB}_{o}-PB}{{PB}_{o}}$$

(2) $$PB=\frac{A_{B}}{A_{B}+A_{R}}$$

## 3. Results and Discussion

### 3.1. Vesicle Size and Size Distribution

Vesicles are typically nanoscopic with diameters ranging from 10 s to 100 s of nanometers. Dynamic light scattering (DLS) was used to measure the size and size distribution of PDA vesicles in solution. Table 2 shows the lognormal, intensity-averaged particle size and dispersity of various polydiacetylene vesicles processed under similar assembly conditions. A sample lognormal distribution and autocorrelation function is provided in the Supplementary Materials (Figure S5). Intensity-averaged particle size is presented over number- or volume-averaged size because it is the metric directly measured by DLS [47]. The primary drawback is that larger particles and aggregates will contribute more to light scattering and skew the data to larger particle sizes. PDA vesicle sizes are often reported as either intensity- or volume-averaged sizes. For all tail lengths, PDA surfactants formed relatively large self-assembled structures (>50 nm), consistent with vesicle assemblies. The diacetylene motif in DA surfactants creates a double kink in the tail, giving them a quasi-two tail lipid structure. This quasi-two tail structure leads to an increased effective volume of the hydrophobic core and slight increase in the molecular headgroup area (~0.25 nm^2^/molecule for PDAs compared to ~0.23 nm^2^/molecule for saturated fatty acids) [48,49,50]. Israelachvili devised a packing parameter relationship to describe how surfactant structure, including the equilibrium area of the head group ($A_{e}$), critical length of the hydrocarbon tail ($l_{c}$), and volume of the hydrocarbon tail ($V_{t}$), gives rise to different self-assembled architectures based on their packing factor, $p_{f}=\frac{V_{t}}{A_{e}l_{c}}$. Assuming a critical hydrocarbon tail length of ~3.2 nm (for PCDA determined by molecular dynamics [51]), a diacetylene spacing between molecules of 0.49 nm (the topochemical requirement for polymerization), and a headgroup area of 0.25 nm^2^/molecule (Langmuir isotherm and XRD data [47,48,52]), $p_{f}\approx 0.75$. This $p_{f}$ corresponds to a truncated cone type packing and the preferred formation of vesicles (${0.5<p}_{f}<1$) [53]. The ratio $l_{c}/V_{t}$ is almost constant, with an increasing number of carbons in the alkyl tail, so the packing factor is mostly affected by the equilibrium headgroup area and alkyl tail width. Thus, vesicles are expected for most commercial PDA surfactant assemblies. Except for the smallest alkyl chain length (TCDA, C23), DLS measurements are consistent with vesicle formation. Future work will use small-angle X-ray and neutron scattering to more accurately characterize the structure of PDA vesicles and validate the lamellarity and thickness of the vesicle bilayer.

Table 2 also shows that PDA vesicle size increases with decreasing alkyl tail length. Short tail PDA surfactants (<C25) were generally unstable and aggregated as is seen with the large and dispersed particle size with TCDA (C23). Attempts to make PDA vesicles of 10,12 heneicosadiynoic acid (ECDA, C21) and octadecadiynoic acid (ODDA, C18) were also unsuccessful and led to near instantaneous aggregation after solvent injection. There are reports in the literature of stabilizing TCDA vesicle formation by tailoring vesicle composition or solution conditions. Still, PDA vesicle formation with carbon tail lengths below C25 is challenging and alternative strategies such as headgroup modification should be considered [25,34,54]. The exact reason for decreased vesicle stability with decreasing tail length is not well understood [22,25,41]. In PDA Langmuir films, domain size increases with decreasing tail length [46]. The results are puzzling as an increased tail length increases attractive van der Waals interactions between the tails (consistent with increased melting temperature of the surfactant). However, the enhanced tail interactions will also increase the bending modulus and the energetic penalty of high curvatures required to form vesicles [55,56]. The rigidity imposed by the diacetylene group may also affect the diacetylene surfactant’s ability to pack and form higher curvatures. In this study the acyl length of the spacer was constant and the alkyl tail after the diacetylene was varied. Vesicle fusion and aggregation during incubation could also be more pronounced in systems with lower melting temperatures leading to large particle sizes, but further studies are required.

The headgroup of the polydiacetylene vesicles also significantly affects their assembly properties and stability. The ethanolamine functionalization of PCDA has been shown to stabilize monolayers at the air–water interface by increasing the hydrogen bonding between the amine and carbonyl groups in the headgroup region [48]. Functionalizing PCDA with ethanolamine slightly decreased the average particle size compared to regular PCDA and allowed for the formation of stable C23 vesicles (Et-TCDA). The synthesis of Et-ECDA (C21) vesicles was also attempted but yielded large aggregate structures. The decrease in stable vesicle size for Et-TCDA and Et-PCDA is hypothesized to be due to the increased hydrogen bonding and closer diacetylene packing. However, the tail length still has a significant role on the PDA vesicle size regardless of headgroup. Polymerized BA-PCDA vesicle solutions turn light blue, indicative of only a small number of polymerized vesicles. The vesicle size was much larger than PCDA or Et-PCDA, but the bulk of the material separated to the air–water interface or crashed out of solution. This is because the bulky aromatic BA-PCDA headgroup decreases solubility and inhibits the high curvature packing necessary for vesicle formation. A differential scanning calorimetry (DSC) of PCDA functionalized with benzoic acid, another headgroup with aromatic interactions, has shown an increase in melting temperature compared to PCDA due to π-π stacking interactions between the headgroups. This is consistent with the difficulty of the BA-PCDA surfactants to pack in an efficient way for vesicle assembly [44]. Dispersities for different DA headgroups are indistinguishable from one another and are considered moderately polydisperse [57]. It is worth noting that many other solvent injection parameters could affect vesicle size and size distribution. Tjandra et al. and Tang et al. thoroughly explored the effects of solvent injection parameters on the size and size distributions of polydiacetylene vesicles [22,23]. Data on the effects of injection concentration on vesicle size for PCDA are given in the Supplementary Materials (Figure S6). The focus here is the effect of surfactant structure on vesicle self-assembly and polymerization.

### 3.2. Polydiacetylene Polymerization Characteristics

#### 3.2.1. Dose Energies of Blue and Red Phase

**Figure 2:**
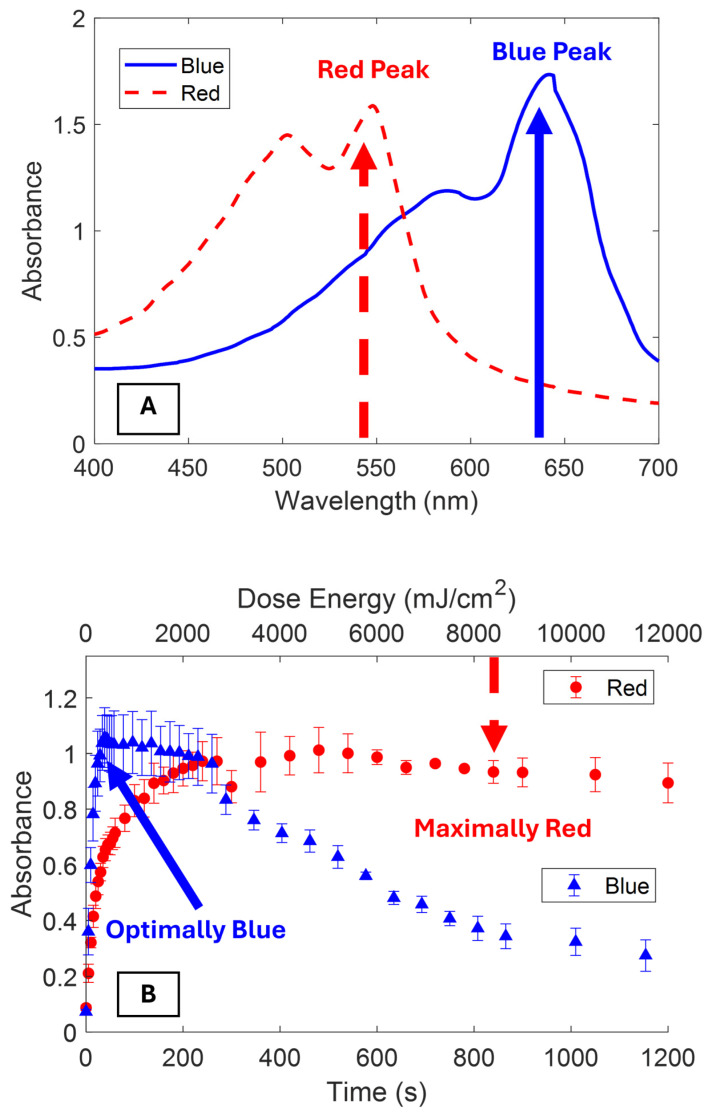
(**A**) Visible absorbance spectra of the blue phase (solid line) and red phase PCDA vesicles. The primary excitonic peaks of the blue (642 nm) and red phases (547 nm) are marked for kinetics studies. (**B**) Evolution of the primary blue (642 nm) and red (547 nm) phase peak absorbance as a function of increasing dose time and energy. The points of optimally blue and maximally red are marked in (**B**) for reference. Error bars are one standard deviation.

Designing effective polydiacetylene sensors requires careful optimization. The goal is to maximize blue phase polymer content while minimizing driving the blue phase into the red phase. The polymerization properties of polydiacetylene vesicles can be characterized by measuring the evolution of the blue (~640 nm) to red phase (~550 nm) with increasing UV dose. Figure 2A shows the absorbance spectra of optimally blue and maximally red PDA vesicles. The blue phase has a primary excitonic peak centered around 640 nm and a vibronic satellite peak around 580 nm. When driven completely into the red phase, the absorption spectrum blue shifts with the excitonic peak at approximately 550 nm and the vibronic peaks at 500 nm. The blue shift is consistent with other reports on the optical properties of polydiacetylene vesicles [4,22,25]. The deconvolution of the primary peaks and additional satellite peaks for the blue and red phases of PCDA can be found in the Supplementary Materials (Figures S7 and S8) along with a table of the relevant peak parameters (Table S1). Figure 2B shows the evolution of the peak blue and red phases as a function of UV dose. The blue phase rapidly increased until reaching a plateau and then slowly transitioned into the red phase. The red phase increased until it reached an asymptotic value. At this plateau, all of the monomer has polymerized and transformed to the red phase. As not all PDAs in the vesicles are aligned for polymerization, the complete polymerization of a sample is not possible. The UV dose energy for optimally blue polymerization was taken as the inflection point of the blue phase where growth was stalled and red phase conversion became dominant over monomer polymerization. The peak red phase was the asymptotic value consistent with the blue phase decaying towards its asymptotic value.

Table 3 shows the dose energies normalized by molar concentration to obtain optimally blue and maximally red vesicles assembled from different monomer surfactant structures (see also Supplementary Figure S9). The UV dose required for maximally red vesicles was 1–2 orders of magnitude greater than that required to polymerize to optimally blue vesicles. In general, the dose energies for optimally blue and maximally red phases increased with increasing tail length, consistent with studies on PDA surfactant vesicles and Langmuir films [25,46]. The increased energy dose is due to increased attractive van der Waals interactions with increased tail length. This increase makes it more difficult for longer tail PDAs to pack in the proper orientation for topochemical polymerization, increasing the energy requirement for polymerization. Polymerized surfactants in the blue phase have a planar backbone. The transition to the red phase is associated with a reorientation of the backbone to a non-planar structure, which changes the conjugation state of the polymer backbone [51,53]. As a result, longer chain length surfactants require higher dose energy when transforming from the blue to red phase. Two notable exceptions to this behavior were BA-PCDA and TCDA. BA-PCDA could not be transformed fully from the blue to red phase even after more than 1 h of UV exposure time. TCDA was not evaluated due to its aggregation and poor vesicle stability.

Substituting the headgroup of the diacetylene surfactant can have wildly different effects depending on the headgroup architecture. When diacetylene surfactants are functionalized with ethanolamine, both polymerization and chromatic transitions are easier and require a lower energy dose. For example, the Et-PCDA energy doses dropped by an order of magnitude, respectively, from PCDA. Other researchers have observed that substituting the fatty acid headgroup with a group that can add hydrogen bonding sites (ethylenediamine or certain amino acids) shifts the thermochromic colorimetric response to lower temperatures and therefore lower energy requirements for the blue to red transition [21,25]. The exact origin of the change in sensitivity is not well understood. One possible explanation is that the additional hydrogen bonding of the headgroup improves the packing state of the surfactants which makes polymerization easier but increases the strain on the polymer backbone. If the strained packing in the monomer state is preserved upon polymerization, this could increase the strain on the polymer backbone and decrease the stability of the blue phase relative to the red. Being able to change and tune the sensitivity is particularly powerful for biosensing applications. As most biological interactions and processes are relatively weak (<15 $k_{b}T$), very sensitive self-assembled PDA systems are needed. To further optimize such systems, future studies with grazing incidence X-ray diffraction can accurately determine the packing state of PDA surfactant films with different headgroups to evaluate their role in packing and backbone strain on the polymer chain.

Functionalizing PDAs with boronic acid had a vastly different effect than ethanolamine. Attempts to self-assemble BA-PCDA vesicles yielded solutions with very low optical densities (low vesicle formation), making them difficult to characterize due to poor signal to noise ratios. The highly polar boronic acid headgroup and phenyl ring accompanied with the long hydrophobic tail cause boronic acid PDAs to segregate to the air–water interface instead of self-assembling in solution. PDA surfactants whose headgroups were substituted with aromatic acids were found to have much higher melting temperatures (>100 °C) due to their additional attractive interaction forces (hydrogen bonding and π-π stacking) [44]. This makes it difficult to heat monomer solutions above the surfactant melting temperature for vesicle formation. Although the optical density was low, BA-PCDA was found to polymerize from blue to a faint purple with increasing UV dose, but never became fully red. This is consistent with other observations of PDAs with aromatic functional groups. For example, PDAs functionalized with polar aromatic compounds like benzoic acid or naphthalene can exhibit chromatic reversibility with temperature stimulus. The blue to red phase thermal reversibility was attributed to enhancements in hydrogen bonding and π-π stacking in the aromatic moieties which make the transition more difficult [42,43]. Reversible PDAs have many interesting applications for real-time, local detection of biological processes that are cyclical, like macromolecule release or the temperature/pH monitoring of live cells. The energy required to polymerize the BA-PCDA to optimally blue is greater than Et-PCDA and less than PCDA. This could be skewed since BA-PCDA preferentially segregated to the air–water interface or crashed out of solution, reducing the concentration of vesicles in solution. The area per molecule of BA-PCDA (~0.27 nm^2^/molecule) is also almost 10% larger than both PCDA and Et-PCDA (both ~0.25 nm^2^/molecule) and could explain the difficulties BA-PCDA has in forming high curvature vesicles [48,49,53].

#### 3.2.2. UV-Induced Reaction Kinetics of PDAs

**Figure 3:**
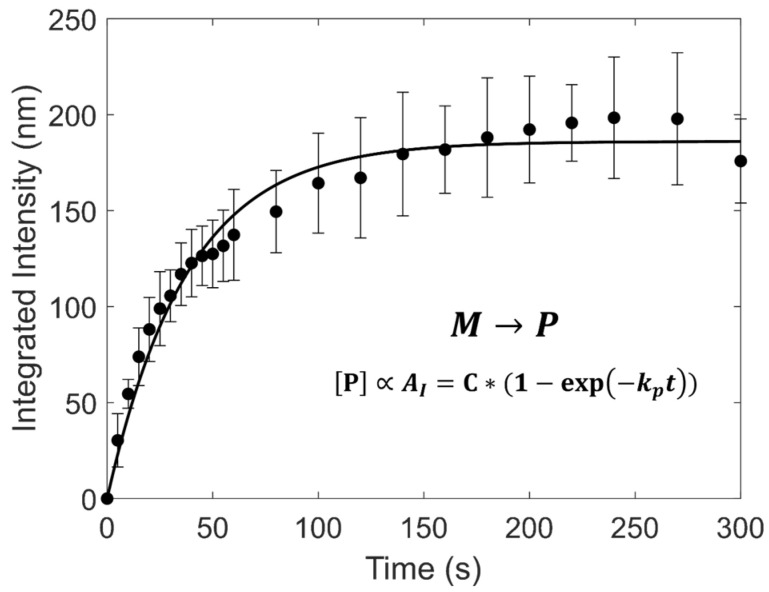
Integrated Intensity as a function of polymerization time for PCDA vesicles fit to a first-order model (R^2^ = 0.92). The fit line is for a first-order polymerization reaction of monomer *[M]* to polymer *[P].* Fitted parameters are listed in Table 4. The UV dose was 9.6 ± 0.9 mW/cm^2^.

Diacetylene monomer (M) polymerization (M→P) is known to follow first-order reaction kinetics. In the case of PCDA Langmuir films, a degradation term is often included to account for photobleaching [58,59]. Photobleaching was not significant in vesicles, likely due to a decrease in the UV–oxygen-induced degradation of surfactants when suspended in water instead of air. If one assumes all of the color in the solution is due to the presence of blue or red polymer, according to Beer’s law, the concentration of polymers is directly proportional to the integrated intensity of the visible spectrum [60]. A sample integrated intensity measurement for PCDA vesicles fit to a first-order reaction model is shown in Figure 3. Integrated intensity data for all vesicle systems used in this study is provided in the Supplementary Materials (Figure S10). Rate constants for the different DAs are reported in Table 4.

**Figure 4:**
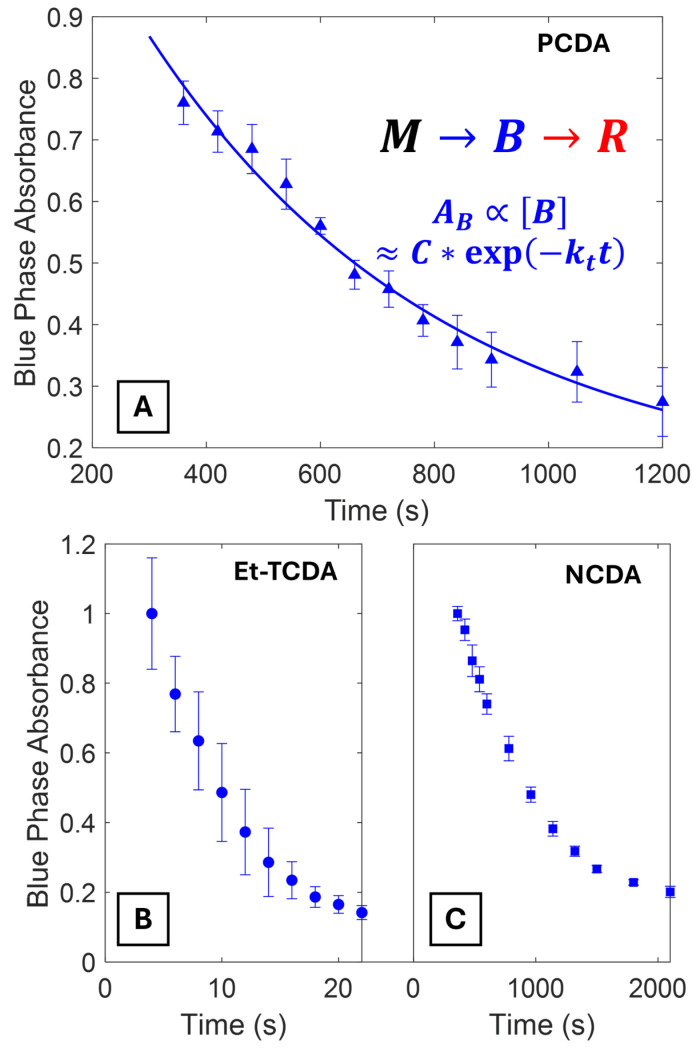
(**A**) Exponential decay of blue phase absorbance for PCDA vesicles at longer timescales. Inset is the chemical reaction between the monomer (M), blue (B), and red phases (R) and the fit equation of the blue phase absorbance (*A_B_*) to an exponential decay model. (**B**,**C**) Blue phase exponential decays for Et-TCDA and NCDA for comparison. Data are normalized to the initial absorbance value to allow direct comparison between different PDAs.

Diacetylene polymerization kinetics are typically much faster than the polydiacetylene blue B to red R phase transition kinetics. In this case, a two-step reaction model is used (M→B→R). The red phase transition rate can be extrapolated by fitting the decay of the blue phase at long timescales to a first-order exponential decay model. Figure 4A shows the blue phase absorbance decay of PCDA vesicles. Figure 4B,C shows two additional PDA vesicle solutions (Et-TCDA and NCDA) with dramatically different decay timescales. Data for other PDA vesicles is provided in the Supplementary Materials (Figure S11).

Table 4 summarizes the measured polymerization and transition rates and their ratios for each surfactant. The polymerization rates were identical and ranged from 0.026 to 0.028 s^−1^ for standard carboxylic diacetylene surfactants with tail lengths from C25 to C29. The transition rate from the blue to red phase showed a modest decrease with increasing tail length. The ratio between the polymerization and transition rates also provides some interesting information on the relative stability and sensitivity of the PDA system. Higher values of k_p_/k_t_ indicate a system that polymerizes but is difficult to transform. In other words, how stable the blue phase is relative to the red phase. This could be important when building vesicle sensors that are less susceptible to transitions due to non-specific interactions.

The substitution of the fatty acid headgroup with ethanolamine drastically increased the polymerization and transition rate of the diacetylene surfactants by about one order of magnitude (e.g., PCDA vs. Et-PCDA). Although TCDA did not form stable vesicles, Et-TCDA did. Ethanolamine functionalization and the shorter chain length resulted in the fastest rate of polymerization and transition. This system polymerizes readily and can transform fully into the red phase in less than 30 s of UV dose. The rapid kinetics is attributed to the interactions of the ethanolamine headgroup favoring the red phase.

Kinetics studies are typically used to extrapolate an activation energy. This is difficult with polydiacetylenes because heating them disrupts the packing required for topochemical polymerization, making it difficult to develop an Arrhenius relation, particularly for polymerization. Previously, Carpick et al. used in situ time-resolved thermal fluorescence measurements to obtain transition kinetics [61]. Understanding the sensitivity of the polymerization and the transition is important for choosing the best sensing system for stability, receptor–ligand bond strength sensitivity, and concentration. The dramatic impact of headgroup modifications using simple peptide coupling schemes provides a powerful tool for self-assembly and tuning polymerization and transition properties.

### 3.3. Thermochromic Transitions

**Figure 5:**
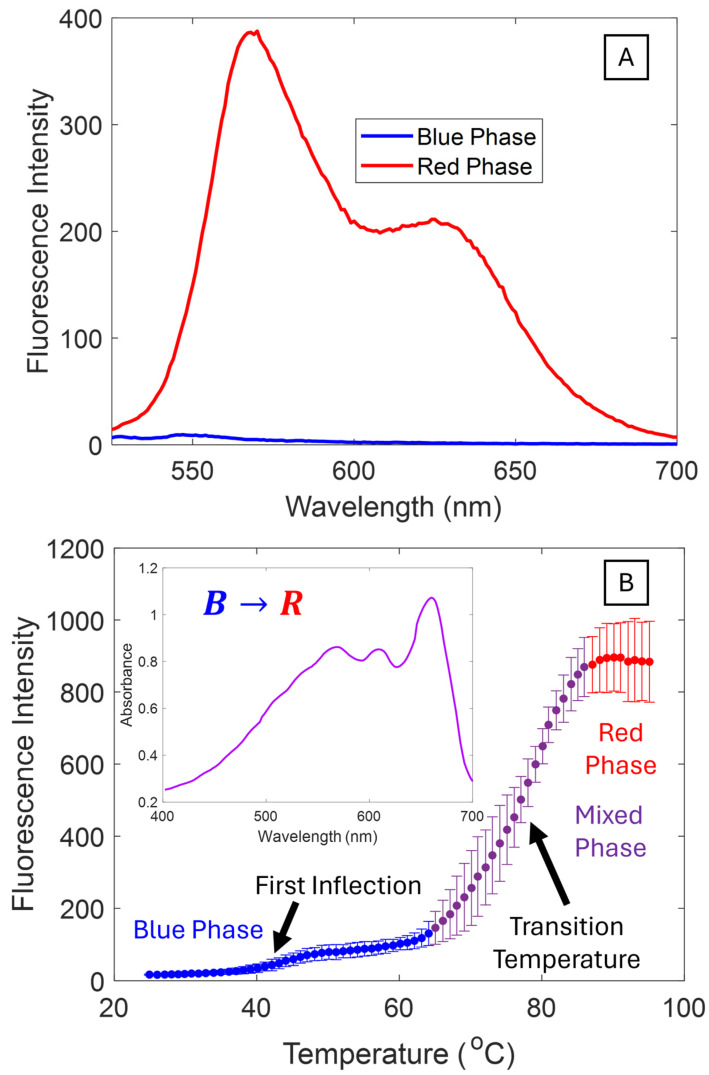
(**A**) Fluorescence spectrum of PCDA blue and red phases. (**B**) Thermal fluorescence curve of PCDA that shows three regions: a blue phase region, a mixed region where the blue phase begins to transform to red, and a plateau region where the system is completely driven into the red phase. Inset is a UV-vis spectra showing the colorimetric properties of the “mixed phase” where there is a mixture of blue and red phases, yielding a purple color.

A common application of PDA sensors is for temperature. PDAs with stronger intermolecular forces transform from blue to red phase at higher temperatures. Thermochromism can be quantified by looking at a change in colorimetric ratio with increasing temperature or directly with fluorescence. Blue PDAs are essentially non-fluorescent while red PDAs are highly fluorescent (Figure 5A). The direct tracking of thermal fluorescence in situ is a convenient means to study the blue to red phase transition. Fits of the fluorescence spectra to Gaussian line shapes for various PDA vesicle solutions are provided in the Supplementary Materials (Figures S12 and S13). Figure 5B shows the intensity of the peak fluorescence of the red phase as a function of temperature for PCDA. Thermal fluorescence spectra of all PDA vesicles explored in this study can be found in the Supplementary Materials (Figure S14). Starting from an optimally blue PDA vesicle solution, three regimes of fluorescence can be observed. In the first regime, the PDAs are primarily blue phase, with minimal background fluorescence coming from background red phase formed during the polymerization. The fluorescence intensity slowly increases with a slight inflection before reaching the transition region where the fluorescence rapidly increases with increasing temperatures. This transition region is often referred to as the “purple” phase—a sometimes reversible intermediary in between the blue and red phases [61]. However, in this case and others (see Supplementary Figure S15A) the transition was not reversible. The fluorescence was maintained after cooling, indicating a permanent transition in the PDA structure. We attribute the inflection to variation in the alkyl tail configuration along the polymerized backbones. Some of the PDAs are slightly further along the reaction coordinate and require lower energies to transform. Similar variations have been observed with the hyperspectral imaging of PDA crystals [62]. Recall that in the blue phase, the alkyl chains are planar and become non-planar in the red phase. In the second regime, the fluorescence increases rapidly with temperature. PDA alkyl side chains have enough thermal energy to allow for the structural reorientation required for the blue to red transition. The inflection point of this transition, where approximately half of the PDAs have transformed from the blue to red phase, is typically taken as the thermochromic transition temperature. At this point, the solution is visibly purple due to the mixture of blue and red phases in solution (Figure 5B inset), not to be confused with a distinct “purple phase” sometimes referred to in the literature [61]. In the third regime, the fluorescence reaches a plateau and all of the blue phase has been transformed into red phase.

Table 5 shows the thermochromic transitions for several PDAs of different tail lengths and headgroups. For Et-TCDA and Et-PCDA, the transition temperature increased with increasing tail length consistent with an increasing difficulty of transition and structural reorientation with increased van der Waals attraction in the tail region. For the fatty acid systems, the same trend is roughly followed. PCDA has the lowest transition temperature (80 °C), while HCDA and NCDA have approximately the same transition temperature (85 °C). The pre-transition region fluorescence intensity increased in extent and slope with temperature. We hypothesize that the distribution of packing geometries, which have different energies for blue to red transition, broadens with increasing chain length. In the case of NCDA, the pre-transition region becomes so broad that there is a decrease in the apparent transition temperature [62].

As expected, substituting the carboxylic acid headgroup with ethanolamine dramatically decreased the transition temperature. The increased hydrogen bonding in the headgroups is thought to increase the strain on the polymer backbone, and thereby lower the energy requirement to transform the vesicles from the blue to red phase. There are several additional features. In the plateau region, there is a decrease in the fluorescence signal that was not observed with their fatty acid vesicle counterparts. This is likely due to fluorescence quenching. There is enough thermal energy for electrons to occupy the excited state, reducing fluorescent events. Et-PCDA systems also did not exhibit a pre-transition region. One explanation is that the increased hydrogen bonding greatly improved the packing order, eliminating different energies of transition. The lack of a pre-transition region is beneficial for building biosensors when an abrupt change in fluorescence state is needed with applied stimuli, such as for a binary switch. The reduced temperature transition, corresponding to an increased sensitivity, is also useful for building sensors for weak bio-chemical and bio-physical interactions like cell tractions or contractions, which are on the order of a mere nN/μm^2^ [46]. The peak fluorescence for the ethanolamine functionalized vesicles is also much higher than the fatty acids, making them particularly powerful for fluorescence-based biosensing systems for increasing signal to noise.

The thermochromic transitions of fatty acid PDAs and ethanolamine functionalized PDAs are irreversible, indicating that the blue state is meta-stable. This limits the sensing applications to binary reporting. Certain PDA vesicle systems with polar aromatic head groups have been demonstrated to exhibit some thermal reversibility. BA-PCDA’s aromatic ring and polar headgroup is similar to the benzoic and napthoic acid that has previously demonstrated reversibility [42,43]. Here, BA-PCDA vesicle transition was not reversible, but we were unable to completely transform the system. Even at 95 °C, BA-PCDA was only partially transformed, and cooling curves revealed a constant fluorescence (Supplementary Figure S15B). Higher temperatures are required to fully drive this system into the red phase. Such high transition energies may be undesirable for biological sensing applications but can likely be modulated by using mixed composition vesicles.

### 3.4. Biosensing with Polydiacetylenes

**Figure 6:**
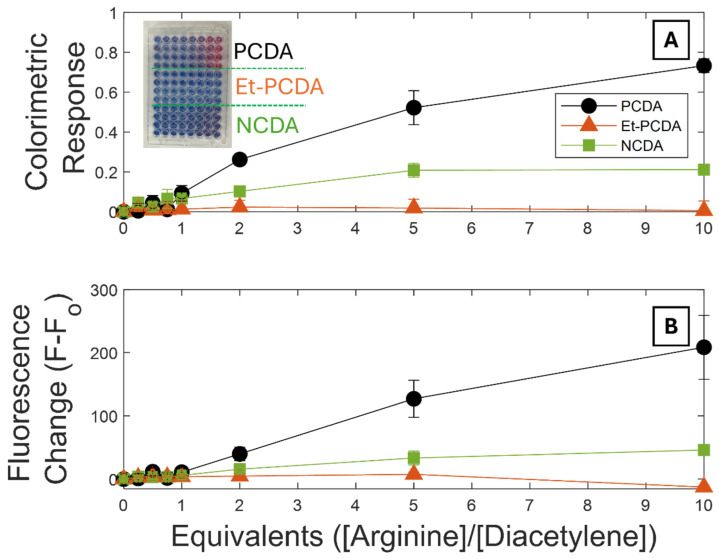
(**A**) Colorimetric response of PCDA, Et-PCDA, and NCDA with molar equivalents of arginine. Colorimetric response measures the consumption of the blue phase (transformation to red phase) with increasing stimuli. (**B**) The fluorescence change from the initial fluorescence value for PCDA, Et-PCDA, and NCDA in response to arginine matches (**A**). Inset is a well plate showing the colorimetric change for all three vesicles. Rows 1–4 show PCDA color change with increasing equivalents of arginine, rows 5–8 show the colorimetric change for Et-PCDA, and rows 9–12 show the colorimetric changes for NCDA.

Since the 1990s, biosensing with polydiacetylenes has primarily been based on receptor–ligand interactions (Table 1). Fatty acid polydiacetylenes have carboxylic acid headgroups which are typically anionic in most physiological aqueous conditions. Due to this negative charge, they have been demonstrated to have transition sensitivity to positively charged amino acids like arginine and lysine without the need for functionalization [63]. Figure 6 shows both a colorimetric response curve (Figure 6A) and fluorescence change curve (Figure 6B) for PCDA, Et-PCDA, and NCDA vesicles exposed to different ratios of arginine. The colorimetric response (CR) measures the decay in the percent blue phase (*PB*) with an applied stimulus. The fluorescence response *(FR)* is simply the difference in fluorescence between the initial and final states. PCDA showed a distinct increase in CR and FR with exposure to increasing concentrations of arginine up to 10 equivalents. NCDA also exhibited a small increase in both CR and FR upon exposure to arginine, though lower in magnitude in comparison to PCDA. This is consistent with the increased van der Waals forces between the tails, and therefore, energy required for the transformation of NCDA. Et-PCDA did not transform upon exposure to arginine, even though both photochromism and thermochromism studies showed Et-PCDA to be more sensitive than PCDA. It can be concluded that the ethanolamine headgroup prevents the adsorption of positively charged amino acids. This points to the important role of multiplexing various PDA vesicles to distinguish between different analytes when designing robust PDA sensing systems.

### 3.5. PDA-Based Biosensors: Future Applications and Outlook

PDAs have been demonstrated as biosensors for both colorimetric and fluorometric sensing applications. Colorimetric-based sensors have the advantage of being visible to the naked eye. Coupling colorimetric responses to spectroscopy enables more quantitative measurements of the color change and concentration analysis of analytes [3,4]. The downside of the colorimetric approach is that it requires more sensor material, longer incubation times, and it is primarily a bulk characterization technique. Local information can be acquired using hyperspectral microscopy at the expense of time and effort [62]. Fluorogenic sensing can be carried out with inexpensive fluorescence microscopy techniques to acquire local information in addition to bulk information from spectroscopy. The higher signal to noise of fluorogenic sensing also opens new opportunities. Examples within a biological context include monitoring cell migration or local receptor–ligand binding events [3,4,13,14,15,16,33,34]. With advances in additive manufacturing, microfluidics, and synthetic biology, PDA sensors with new features such as sensitivity gradients or multi-mode biological sensing can be designed.

The PDA biosensing community has exploded over the years. The quantitative usage of PDAs has primarily focused on detecting the concentration of biological analytes using a colorimetric response. The bio-specific receptor–ligand interaction binding event perturbs the PDA backbone and drives the PDA transition from blue to red. However, there are many opportunities to improve the quantitative nature of PDA sensing. Biological interactions are often force/energy specific and the summation of the binding events on the vesicle must be sufficient to drive the transition from blue to red. Direct force measurements of PDA transitions are challenging and are only recently being discovered using surface force apparatus and atomic force microscopy [18,64,65]. In the future, an improved understanding of how mechanical forces drive these transitions may be useful for certain biological interactions. PDAs can be used in conjunction with other biophysics techniques that measure forces between receptors and ligands such as surface force apparatus, atomic force microscopy, or optical tweezers to quantify the force and energy requirements of the transition [66,67,68].

In addition to studying receptor–ligand interactions more quantitatively, a future focus for PDA vesicle biosensing is fluorescence and confocal fluorescence microscopy for the quantitative exploration of cellular processes. In a biophysical context, PDAs might be used to detect mechanical cellular processes like growth, migration, or contraction under in vitro conditions. Finney et al. explored the usage of two-dimensional (2D) polydiacetylene Langmuir films to measure slime mold migration and cell traction stresses. The use of vesicles could enable 3D biosensing platforms [18]. For example, vesicle sensors can be loaded into a hydrogel along with cells to passively and locally investigate cell growth or migration. The vesicles that transform would provide a local measurement of cell activities via fluorescence microscopy. If the PDA vesicles were calibrated, an array of vesicles with different sensitivities could measure the deformations applied by cells in 3D hydrogels. Multi-dimensional biochemical systems can also be designed for cells where vesicles can be tagged with ligands that respond to cell-secreted molecules. These can be utilized to quantitatively study cell secretion rates of macromolecules, cations or other biochemical processes under physiological or disease conditions.

Reversible polydiacetylenes have potential interest for studying the cyclical processes of cells. In general, the PDA transition is non-reversible. As a result, PDA sensors can only “sense” a singular event. Researchers have designed PDAs whose color change is reversible by increasing the interaction strength of the head group region to stabilize the blue phase over the red phase [42,43,45]. One way to do this is through the PDA fabrication in the presence of a divalent heavy metal cation. Zn^2+^, which forms a bridging bidentate between the carboxylic acid headgroups of neighboring surfactant molecules, is especially effective [39,46]. Another method is to functionalize the head group with polar aromatic moieties, which add additional hydrogen bonding and π-π stacking interactions. These approaches can work well for colorimetric sensors, but less so for fluorogenic sensors because the cations quench fluorescence and the aromatic polydiacetylenes are much more weakly fluorescent. Future work should focus on designing PDA surfactant systems that are strongly fluorescent and reversible. This could potentially be done using a Forester Resonant Energy Transfer (FRET) approach where a resonant energy transfer dye can be mixed with the blue phase PDA or tagged to the surfactant [69]. If the reversible system is weakly fluorescent, the change in color from blue to red can drive an off–on response in the loaded fluorophore rather than the PDAs themselves, boosting the fluorescent response for sensing.

## 4. Conclusions

In this work, PDA vesicles were synthesized with varying alkyl tail lengths and head group architectures to evaluate their assembly, polymerization, and transition properties. PDAs with increasing tail lengths were generally found to require more energy for polymerization and transition due to increased van der Waals interactions between packed tails. The increase in thermochromic transition temperatures with tail length is well established. PDA surfactants with tail lengths below C25 were difficult to work with and did not appreciably produce vesicles. Instead of decreasing tail length to make more sensitive biosensors, PDA users should consider headgroup modification for increasing vesicle transition sensitivity. For example, ethanolamine functionalization significantly decreased the polymerization energies, transition energies, and thermochromic transition temperature of PDA. Ethanolamine functionalization also enabled the formation of stable C23 PDA vesicles. These systems were also found to be non-responsive to arginine and could be used in sensing systems avoiding non-specific interactions. Conversely, boronic acid headgroup functionalization inhibited vesicle self-assembly and transition to the red phase. The strong π-π stacking and hydrogen bonding interactions in the headgroups, as well as steric interactions, make the transition from the blue to red phase more difficult. In terms of biosensing performance, PCDA and NCDA were found to have increasing responsivity to arginine with decreasing hydrocarbon tail length, while ethanolamine functionalization prevented binding and sensor response. Future work for amino acid sensing could focus on tailoring the sensitivity of multiple formulations of PDA vesicles to sense all 20 amino acids. Multiplexing with different PDA vesicle assays can be designed to differentiate the presence of various amino acids in an analyte of interest. PDA sensors are inexpensive, and detection can be either colorimetric or fluorometric. The strength of surfactant-based PDA biosensors is their ability to self-assemble, commercial availability, and ease of chemical functionalization with peptide coupling reactions. These features provide a wide parameter space for researchers to explore and tailor PDA to meet their sensing needs. One area that has been underexplored is mixed PDA systems that tune the ratio of functionalized to non-functionalized surfactants to enhance specificity and sensitivity. Researchers have only scratched the surface of building custom PDA biosensing platforms.

## Figures and Tables

**Table 1:** Brief Review of Different PDA Biosensor Applications and Device Designs.

System	Mode of Sensing	Application	Transformation Characterization	Reference
PDA Langmuir Blodgett Bilayer on Glass	Sialic Acid– Hemagglutinin Binding	Influenza Detector	UV-Vis Spectroscopy/ Colorimetric Response	Charych et al. [3]
PDA-Peptide Amphiphiles Fibers	Cell–RGD peptide Binding	Cell Adhesion	UV-Vis Spectroscopy	Ramakers et al. [13]
PDA Liposomes with Phenyl- Boronic Acid	Sialic Acid– Boronic Acid Binding	Cell Surface Imaging	UV-Vis Spectroscopy/ Fluorescence Spectroscopy/ Fluorescence Microscopy	Wang et al. [14]
Guanidinium-PDA Nanosheets	LPA–PDA–Gu Binding	Ovarian Cancer Detector	UV-Vis Spectroscopy/ Fluorescence Spectroscopy	Hu et al. [15]
PDA Vesicles Patterned to Silicon	EDEA/EDA– α-Cyclodextrin Binding	α-Cyclodextrin Detector	Fluorescence Microscopy/UV-Vis Spectroscopy	Kim et al. [16]
PDA Grafted to PDMS Microfluidic Device	Compression/ Shear Stress	Microalgae Shear Sensor	Fluorescence Microscopy	Casimiro et al. [17]
Supported PDA Langmuir Films	Normal Load/Shear Stress	Slime Mold Traction Sensor	Surface Force Apparatus/ Fluorescence Microscopy	Finney et al. [18]

**Table 2:** Summary of particle size and dispersity of surfactants utilized in this study.

Head Group	Tail Length	Effective Diameter (nm)	Dispersity
Carboxylic Acid	C23	1400 ± 200	0.30 ± 0.02
	C25	140 ± 20	0.22 ± 0.05
	C27	80 ± 20	0.28 ± 0.06
	C29	70 ± 10	0.31 ± 0.02
Ethanolamine	C23	240 ± 40	0.25 ± 0.04
	C25	80 ± 20	0.27 ± 0.03
Boronic Acid	C25	340 ± 30	0.23 ± 0.02

**Table 3:** Summary of respective dose times and energies required to polymerize all relevant surfactants to their optimally blue and maximally red phases.

Surfactant	Optimal Blue Phase Dose Energy (Jcm^−2^ mM^−1^)	Optimal Blue Phase Time (s)	Maximal Red Phase Dose Energy (Jcm^−2^ mM^−1^)	Optimal Red Phase Time (s)
Et-TCDA	0.16 ± 0.01	2.6 ± 0.6	1.7 ± 0.4	28 ± 6
Et-PCDA	1.1 ± 0.1	17 ± 3	14 ± 3	220 ± 40
PCDA	3.9 ± 0.5	70 ± 10	100 ± 30	1900 ± 400
HCDA	4.6 ± 0.7	77 ± 9	140 ± 20	2300 ± 300
NCDA	5.3 ± 0.7	84 ± 6	170 ± 30	3100 ± 200
BA-PCDA	2.2 ± 0.2	28 ± 4	N/A	N/A

**Table 4:** Summary table of all the polymerization rates (k_p_), transition rates (k_t_), and the ratio between the two for PDA vesicle solutions.

Surfactant	k_p_ (1/s)	k_t_ (1/s)	k_p_/k_t_
Et-TCDA	0.53 ± 0.03	0.13 ± 0.03	4 ± 1
Et-PCDA	0.10 ± 0.01	0.017 ± 0.003	6 ± 1
PCDA	0.026 ± 0.002	0.0019 ± 0.0004	14 ± 3
HCDA	0.028 ± 0.002	0.0016 ± 0.0002	18 ± 3
NCDA	0.027 ± 0.001	0.0014 ± 0.0002	19 ± 3

**Table 5:** Thermochromic transitions of the onset, transition, and saturation temperature for PDA vesicle solutions.

Surfactant	Onset Temperature (°C)	Transition Temperature (°C)	Saturation Temperature (°C)
Et-TCDA	44	50	56
Et-PCDA	55	62	68
PCDA	72	80	87
HCDA	80	85	89
NCDA	77	85	92

## Data Availability

Data not in an open repository.

## Supplementary Materials

The following supporting information can be downloaded at https://www.mdpi.com/article/10.3390/bios15010027/s1. Figure S1. NMR Spectra of Et-PCDA. Figure S2. NMR Spectra of Et-TCDA. Figure S3. Fluorescence Spectra of BA-PCDA bound to alizarin red. Figure S4. Schematic of Polymerization Mechanism of PDA’s and the in lab polymerization schematic. Figure S5. Lognormal and cumulative particle size distributions for PCDA and its corresponding correlation function. Figure S6. Deconvoluted spectra of blue and red Phase PCDA. Table S1. Fitted parameters for peaks of deconvoluted blue and red phases of PCDA. Figure S7. Blue and red phase spectra of all PDA’s used in this study. Figure S8. Photochromism plots of all PDA’s used in this study. Figure S9. Integrated intensity curves of all PDA’s used in this study. Figure S10. Blue phase decay curves of all PDA’s used in this study. Figure S11. Deconvolution of the fluorescence spectra of PCDA. Figure S12. Red phase fluorescence spectrum of all PDA’s used in this study. Figure S13. Thermal fluorescence profiles of all PDAs used in this study. Figure S14. Reversible Thermal fluorescence plots for PCDA at the pretransition range and BA-PCDA. Ref. [70] is cited in the Supplementary Materials.
