# Supplementary material for: Fluorogenic Biosensing with Tunable Polydiacetylene Vesicles
Source: Biosensors (Basel). 2025 Jan 7;15(1):27. doi: 10.3390/bios15010027 (PMC11763271; doi:10.3390/bios15010027)
Supplement: Supplementary file 1 [file biosensors-15-00027-s001.zip › biosensors-3314030-supplementary.pdf]

Supporting Information for article:

Fluorogenic Biosensing with Polydiacetylene Vesicles: Tunability and Sensitivity

John S. Miller[a,b], Tanner J. Finney[b,c], Ethan Ilagan[b], Skye Frank[b], Ye Chen-Izu[def], Keishi Suga[g], and Tonya L. Kuhl[b]

[a]Department of Materials Science and Engineering, University of California Davis, Davis, CA

[b]Department of Chemical Engineering, University of California Davis, Davis, CA

[c]Materials Synthesis and Integrated Devices, Materials Physics and Applications Division

Los Alamos National Laboratory, Los Alamos, New Mexico, USA

[d]Department of Biomedical Engineering, University of California Davis, Davis, CA

[e]Department of Pharmacology, University of California Davis, Davis, CA

[f]Department of Internal Medicine/Cardiology, University of California Davis, Davis, CA

[g]Department of Chemical Engineering, Graduate School of Engineering, Tohoku University, 6-6-07 Aoba, Aramaki-aza, Aoba-ku, Sendai, Miyagi 980-8579, Japan

*Correspondence: tlkuhl@ucdavis.edu

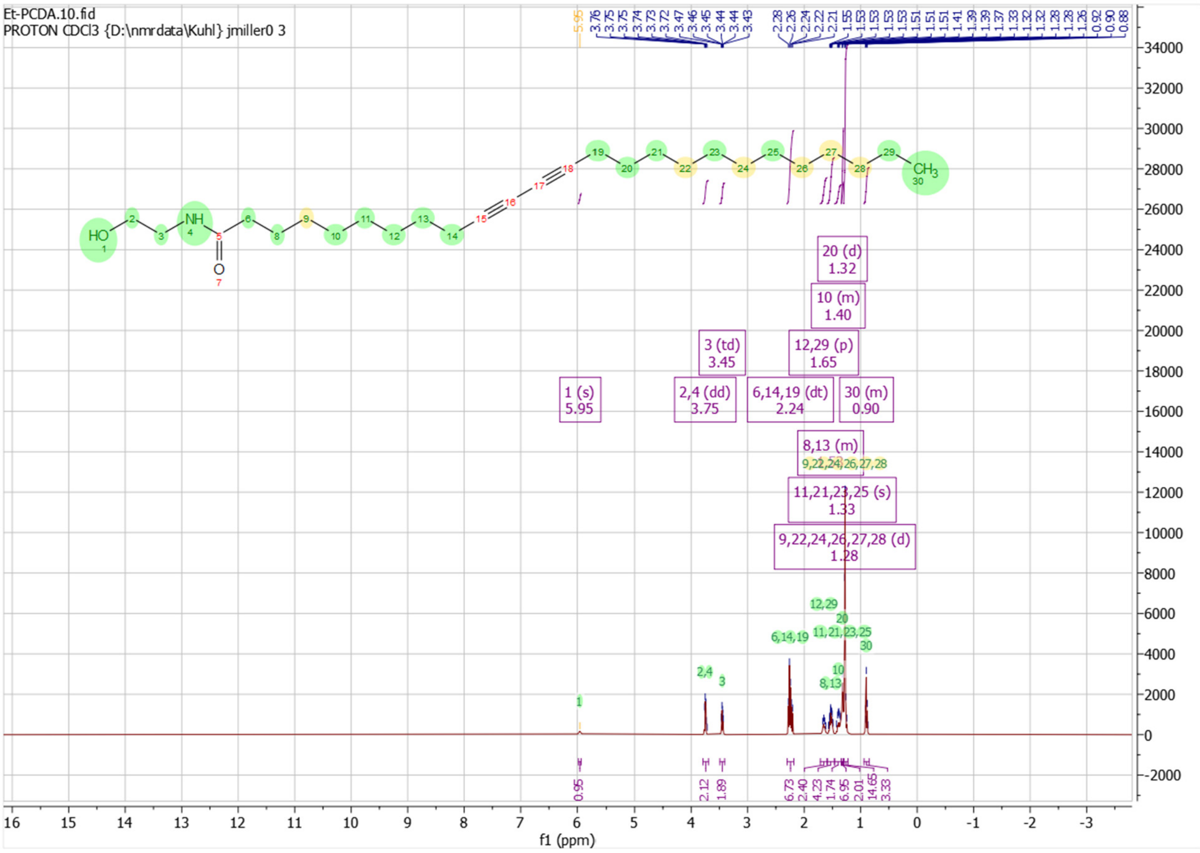

Figure S1. Proton NMR Spectra of ethanolamine functionalized PCDA in deuterated chloroform. The amine peak is inset and blown up due to its small intensity compared to other peaks. The frequency of the NMR was 400 Hz.

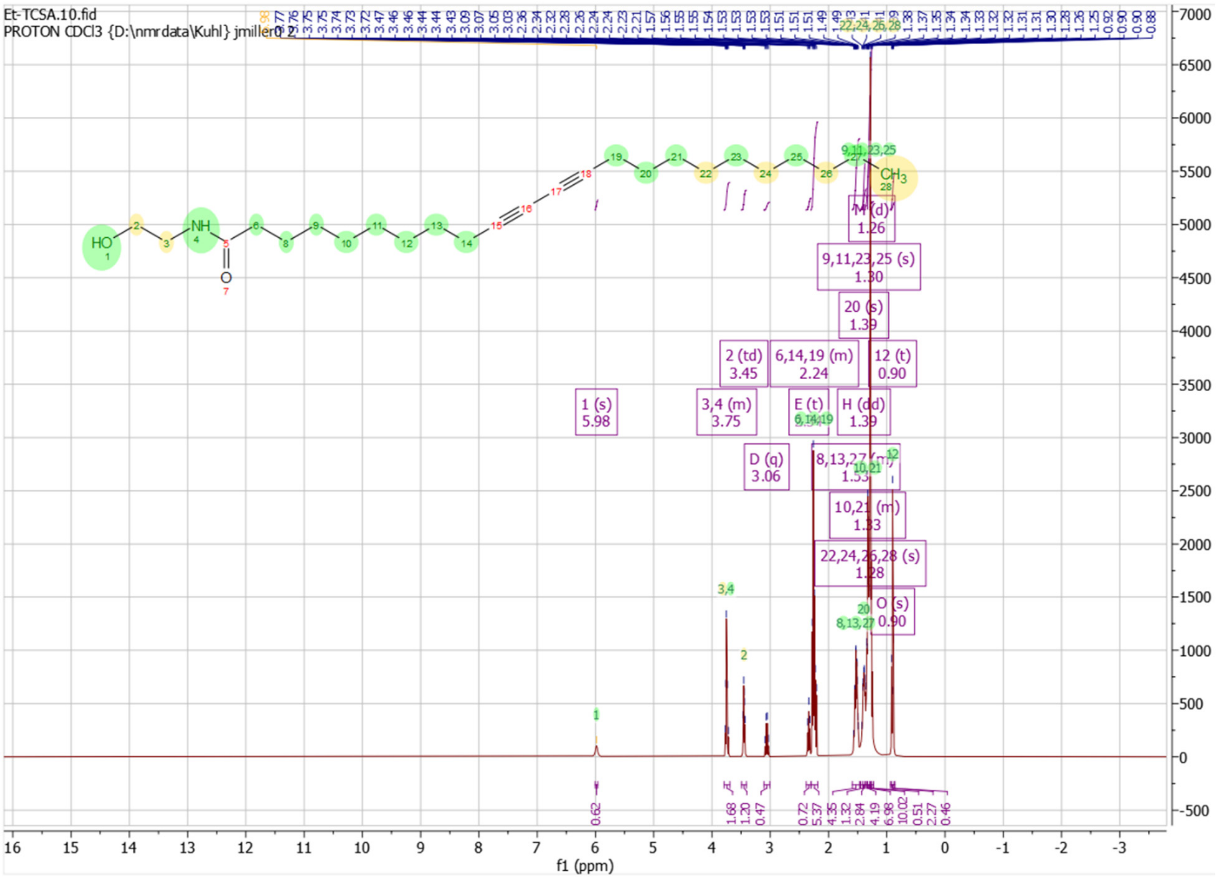

Figure S2. Proton NMR Spectra of ethanolamine functionalized TCDA in deuterated chloroform. The amine peak is inset and blown up due to its small intensity compared to other peaks. The frequency of the NMR was 400 Hz.

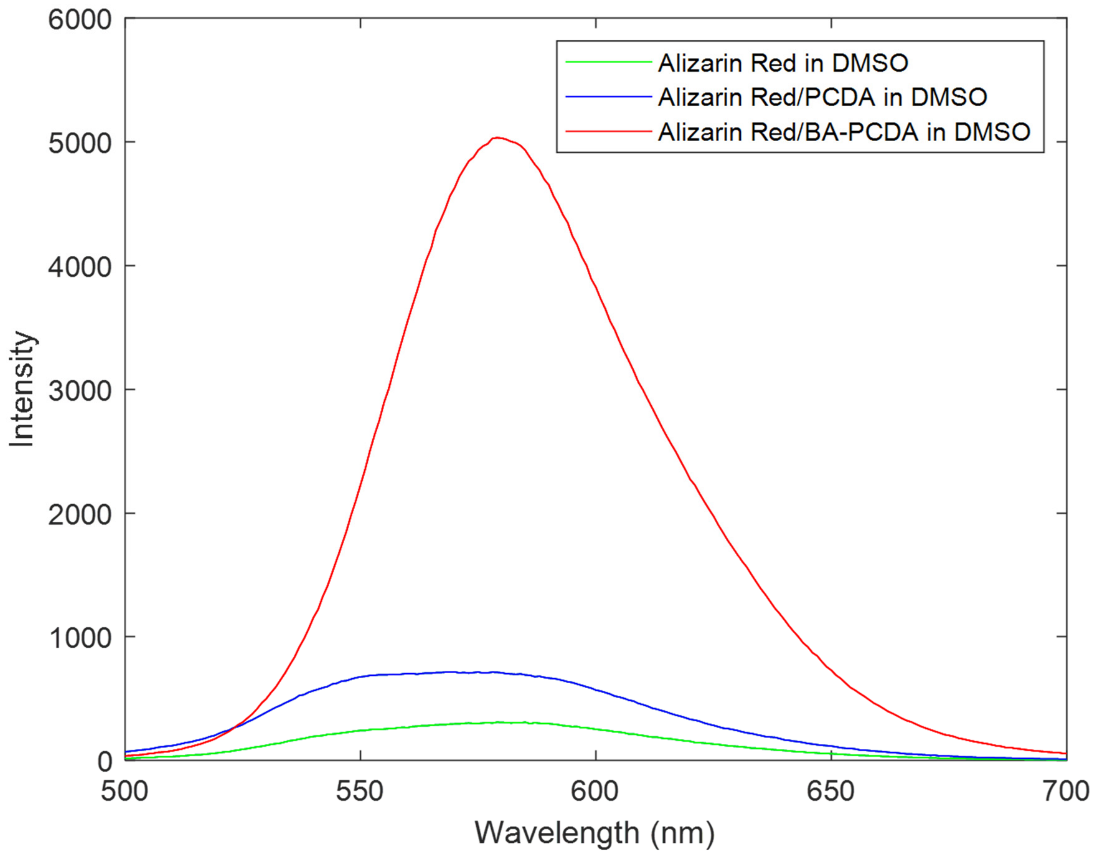

Figure S3. Fluorescence spectra of Alizarin red dye dissolved in dimethyl sulfoxide (DMSO) and mixed with BA-PCDA (red) and PCDA (blue). A reference where the free Alizarin Red unmixed in solution is also shown in green. Alizarin red becomes fluorescent upon binding with boronic acid species [1]. Fluorescence enhancement of BA-PCDA surfactants verifies successful synthesis of a BA-PCDA structure. PCDA also results in some slight fluorescence increase which is still insignificant relative to the BA-PCDA fluorescence enhancement.

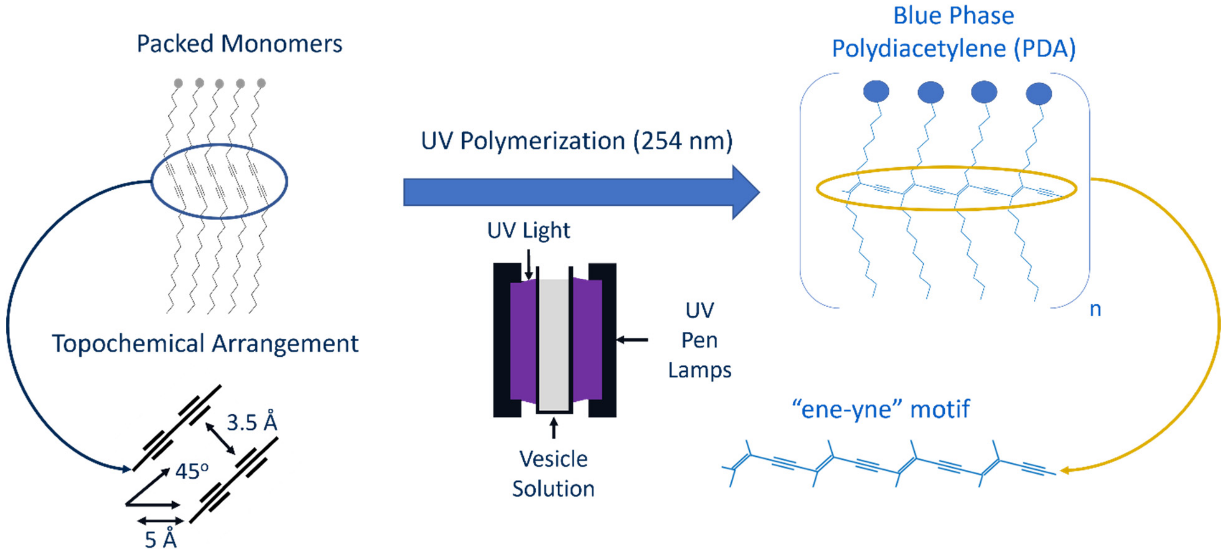

Figure S4. Schematic that shows the work flow for polymerization of vesicles. Monomers self-assembled into vesicles arrange in the proper topochemical arrangement to enable polymerization. Vesicle solutions are polymerized between two pen lamps (254 nm UVC) until the desired polymerization extent is achieved. The result is a visibly blue phase polymer with an alternating double-bond triple-bond motif ("ene-yne") along the polymer backbone.

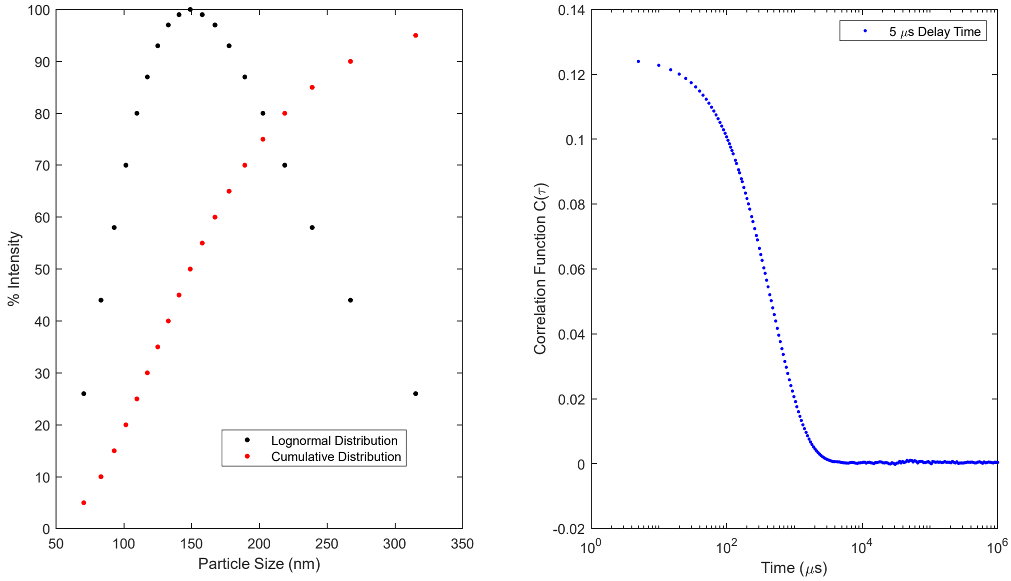

Figure S5. (Left) Representative lognormal and cumulative distribution functions for a sample of PCDA vesicles and (Right) correlation function for a PCDA sample.

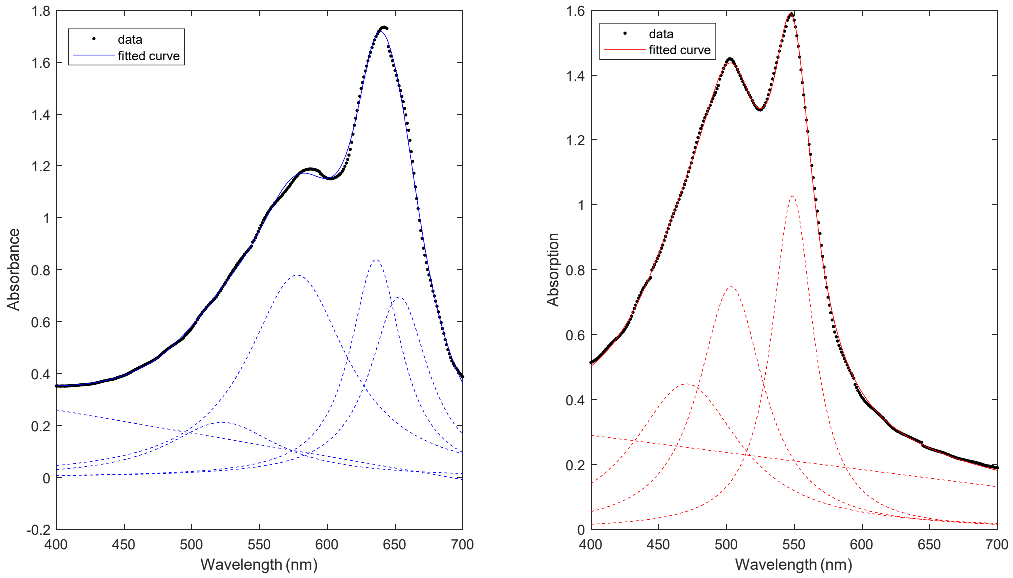

Figure S6. (Left) Blue PCDA Spectra and (Right) red PCDA Spectra with peaks deconvoluted using Lorentzian functions.

Table S1. Relevant peak fit parameters blue and red phase peaks for PCDA

Phase	Peak	Peak Wavelength (nm)	FWHM (nm)
Blue	Peak 1	522 +/- 4	100 +/- 9
	Peak 2	577.8 +/- 0.7	89 +/- 5
	Peak 3	636 +/- 2	46 +/- 4
	Peak 4	653 +/- 3	53 +/- 4
Red	Peak 1	548.83 +/- 0.08	37.5 +/- 0.3
	Peak 2	503.5 +/- 0.5	58 +/- 2
	Peak 3	471 +/- 3	96 +/-7

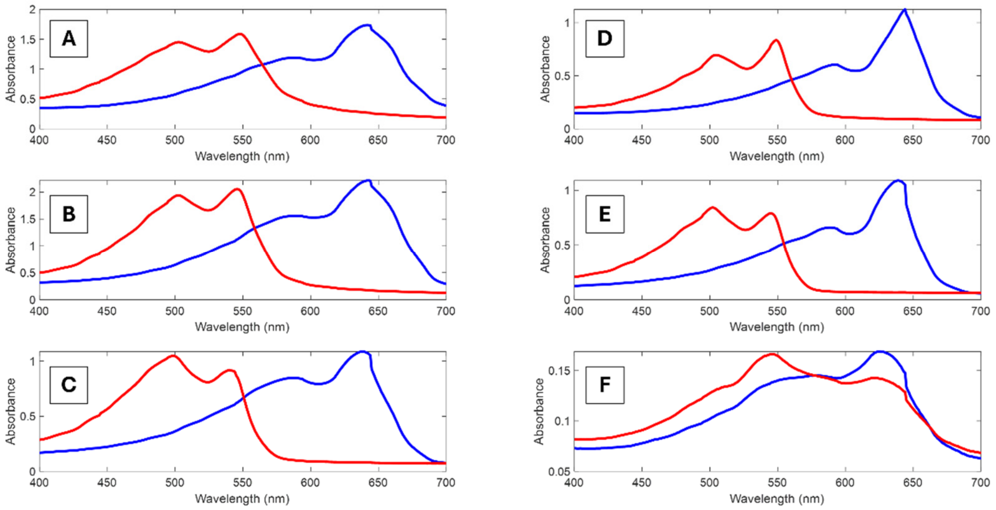

Figure S7. Blue and red phase spectra for (A) PCDA, (B) HCDA, (C) NCDA, (D) Et-TCDA, (E) Et-PCDA, and (F) BA-PCDA. Blue phase PDA spectrum was obtained by polymerizing each system to its optimally blue form. Red phase spectrum is acquired by thermally transitioning the optimally blue phase vesicles into the red phase by heating the solutions up to 95°C. BA-PCDA was unable to fully transition to the red phase, and a proportion of the blue phase remains in the system.

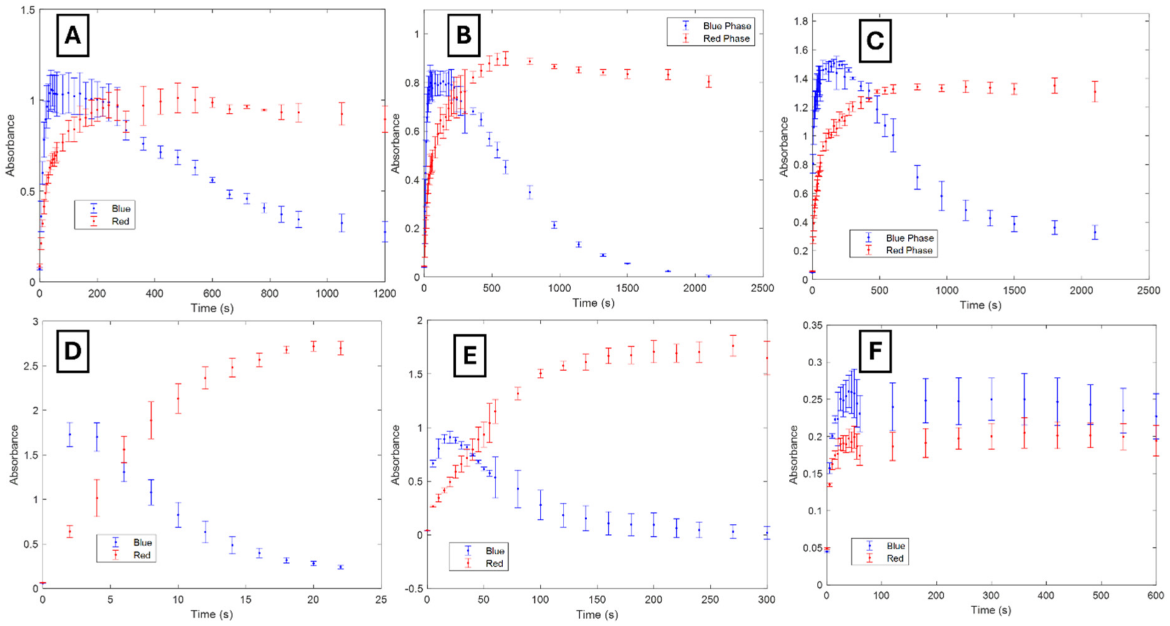

Figure S8. Photo-chromism plots of all systems utilized in this study including: (A) PCDA, (B) HCDA, (C) NCDA, (D) Et-TCDA, (E) Et-PCDA, and (F) BA-PCDA. The nominal blue peak (~640 nm) and red peak (~550 nm) is tracked for each system as a function of increasing UV-dose (9.6±0.9 mJ/cm^2). A minimum of 3 samples were performed for each kinetic study. Sample fractions vary from 12-36 depending on the UV-dose required to fully evaluate the optimally blue and maximally red portions of the curve. Multiple rows of a well plate were utilized and a linear smoothing algorithm was employed on datasets where a new row was utilized to account for instrument errors from moving to different rows of the well plate or separate well plates entirely. The dose time and energy to the optimally blue phase was determined by the peak of the blue absorbance as a function of increasing UV-dose. The dose time and energy of the maximally red phase is determined by the decay of the blue phase when it decays 97.5% of its infinite absorbance value.

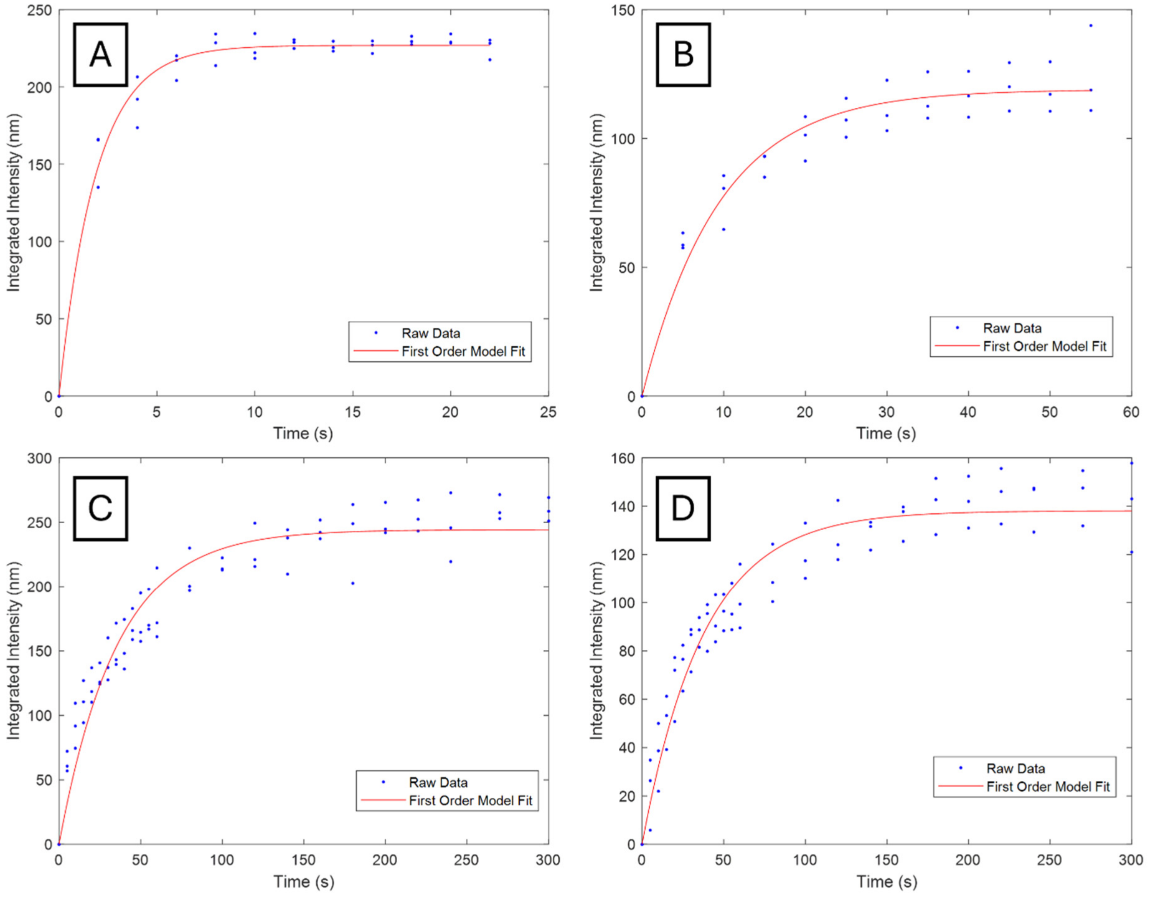

Figure S9. First order fits of integrated intensity curves as a function of UV-dose for (A) Et-TCDA, (B) Et-PCDA, (C) HCDA, and (NCDA). Functions were fit with the following equation: II=A(1-exp(-k_pt)) using non-linear least squared regression in MATLAB. Here t is the dose time, k_p is the polymerization rate, and A is a proportionality constant related to the initial monomer content in solution and absorbance constant of the PCDA solution which relates the integrated intensity to the polymer concentration in solution. R^2 values for all samples were above 0.90. All data points were subtracted from the initial integrated intensity with no UV-dose which serves as the background.*

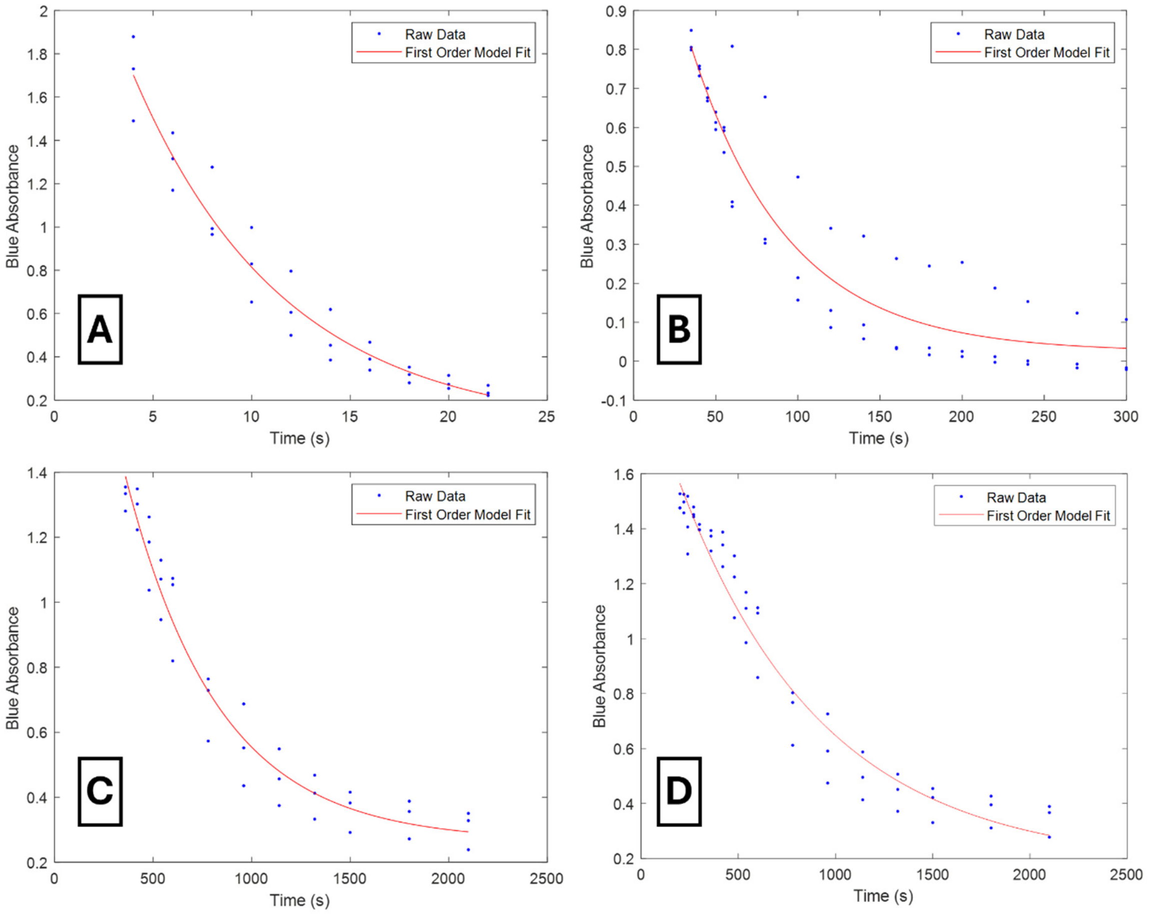

Figure S10. First order decay model fit to the blue phase with increasing UV-dose time for relevant diacetylene surfactants utilized in this study including: (A) Et-TCDA, (B) Et-PCDA, (C) HCDA, and (D) NCDA. Only the tail end of the diacetylene surfactants were fit corresponding to the region where all of the monomer was consumed and $k_p[M]$~0. Data was fit in MATLAB to the following function: I_B=Aexp(-k_tt)-C using non-linear least squared regression and R^2 values range from 0.88-0.96. In the fitting equation k_t is the transformation rate, A is a proportionality constant related to the initial monomer concentration and the Beers law absorbance constant, and C is an offset constant which considers an exponential decay to a non-zero value.

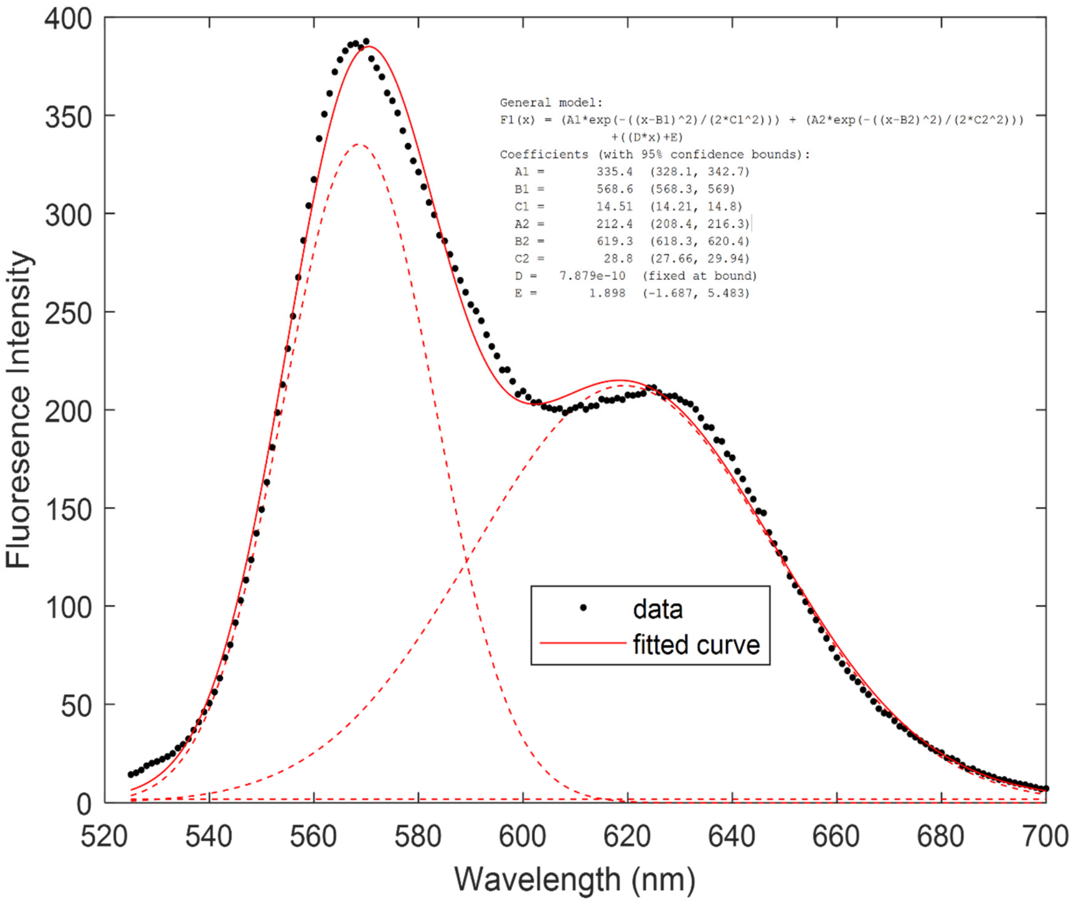

Figure S11. Deconvolution of the fluorescence spectrum of red phase PCDA into two Gaussian peaks. Fit parameters are inset into the figure.

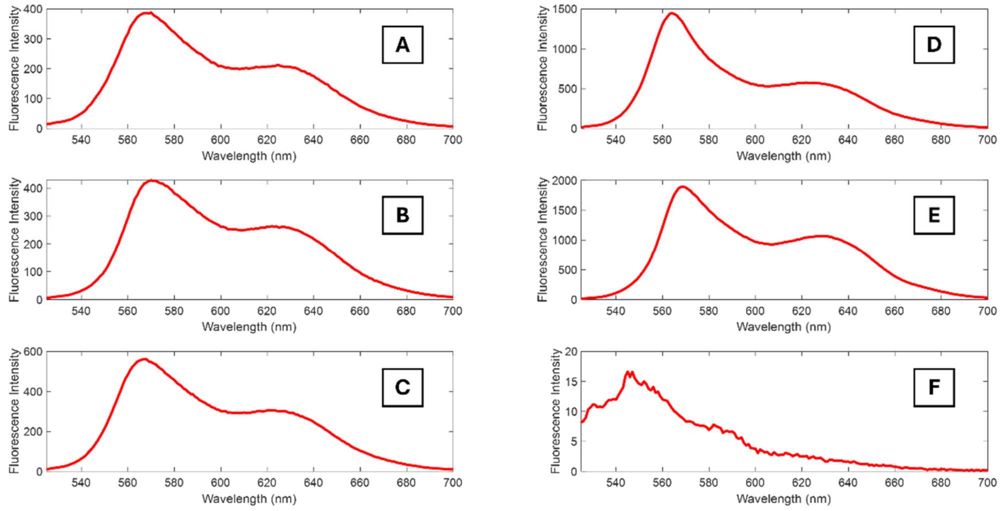

Figure S12. Red phase fluorescence spectrum for all polydiacetylenes investigated in this study including: (A) PCDA, (B) HCDA, (C) NCDA, (D) Et-TCDA, (E) Et-PCDA, and (F) BA-PCDA.

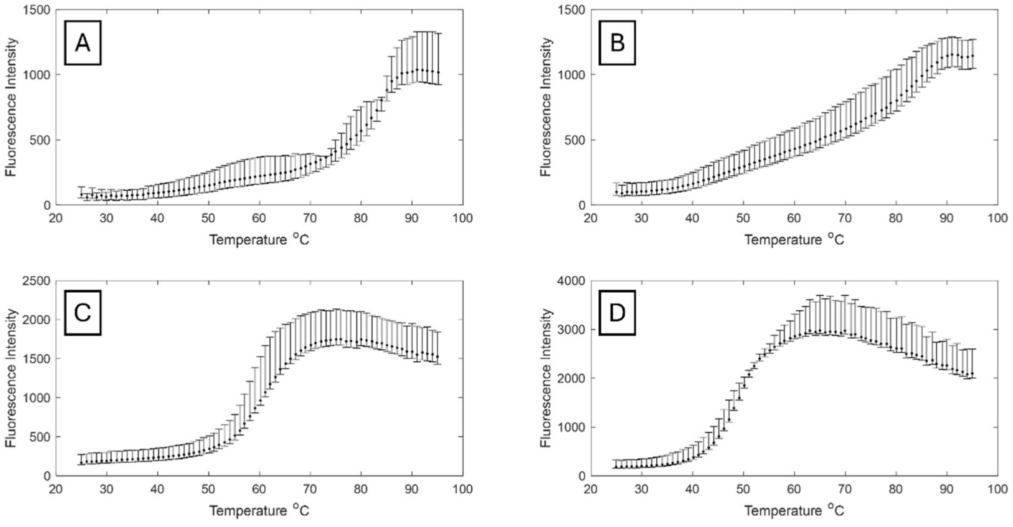

Figure S13. Thermal fluorescence of relevant vesicles studied in this work including (A) HCDA, (B) NCDA, (C) Et-TCDA, and (D) Et-PCDA. Thermal chromic transitions were determined using the intersecting line method where the transition region was fit to a line. The onset and completion of the transition are marked when the temperature data deviated by more than 5% of the linear fit region. The transition temperature was then defined as the midpoint between the onset and completion temperatures.

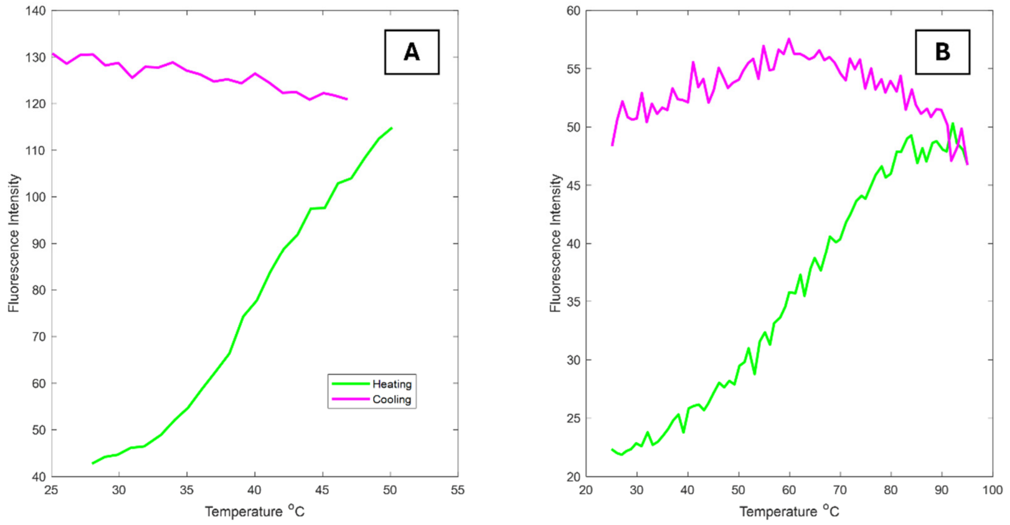

Figure S14. (A) Heating and cooling curves of PCDA vesicles heated up to 50°C and (B) BA-PCDA vesicles heated up to 95°C.

[1] Tomsho, J. W.; Benkovic, S. J. Elucidation of the Mechanism of the Reaction between Phenylboronic Acid and a Model Diol, Alizarin Red S. *J. Org. Chem.* **2012**, *77* (5), 2098–2106. https://doi.org/10.1021/jo202250d.
